# Dipeptidyl Peptidase 4 Activity Is Related to Body Composition, Measures of Adiposity, and Insulin Resistance in Subjects with Excessive Adiposity and Different Degrees of Glucose Tolerance

**DOI:** 10.1155/2019/5238013

**Published:** 2019-02-11

**Authors:** Wellington S. Silva Júnior, Maria das Graças C. Souza, José Firmino Nogueira Neto, Eliete Bouskela, Luiz Guilherme Kraemer-Aguiar

**Affiliations:** ^1^Postgraduate Program in Clinical and Experimental Physiopathology (FISCLINEX), State University of Rio de Janeiro (UERJ), Rio de Janeiro, RJ 20551-030, Brazil; ^2^Endocrinology Discipline, Faculty of Medicine, Federal University of Maranhão (UFMA), Pinheiro, MA 65200-000, Brazil; ^3^Laboratory for Clinical and Experimental Research on Vascular Biology (BioVasc), UERJ, Rio de Janeiro, RJ 20550-013, Brazil; ^4^Laboratory of Lipids (LABLIP), UERJ, Rio de Janeiro, RJ 20551-030, Brazil; ^5^Endocrinology, Department of Internal Medicine, UERJ, Rio de Janeiro, RJ 20551-030, Brazil

## Abstract

**Background:**

The enzyme dipeptidyl peptidase 4 (DPP4) has been recently recognized as an adipo-myokine. However, studies that associate its constitutive activity with body composition, anthropometry, and insulin resistance (IR) are very scarce and included only healthy people.

**Methods:**

First, we investigated the relationships of constitutive DPP4 activity, body composition (assessed by bioelectrical impedance analysis), and measures of adiposity and IR in fifty-two subjects of both sexes, 18-50 years, and BMI ≥25.0 kg/m^2^ who comprised three groups according to glucose tolerance. Additionally, we evaluated associations among DPP4 activity and adipokines, gut peptides, and biochemical variables at fasting and 30 and 60 min after a standardized meal intake.

**Results:**

DPP4 activity was no different among the three groups. At fasting, pooled analysis showed it was positively correlated with measures of central adiposity, such as WC (*P* = 0.011) and WHR (*P* = 0.009), and with all measures of IR, but inversely related to indexes of general adiposity, such as fat mass percentage (*P* = 0.014) and BAI (*P* = 0.0003). DPP4 activity was also associated with lean mass (*r* = 0.57, *P* < 0.0001). After meal intake, DPP4 activity remained significantly associated with insulin, leptin, and resistin. In multiple regression analysis, BAI, WHR, percent lean mass, HOMA-IR, and leptin influenced DPP4 activity and explained approximately 26% of the variance on it.

**Conclusions:**

Constitutive DPP4 activity is positively associated with lean mass, central adiposity, and IR and negatively to general adiposity. Furthermore, it seems to be influenced by body composition and IR and could also be viewed as an adipo-myokine in subjects with excessive adiposity and different stages of glucose tolerance.

## 1. Introduction

Dipeptidyl peptidase 4 (DPP4), also known as adenosine deaminase-binding protein or cluster of differentiation 26 (CD26), is a serine protease widely expressed by many specialized cell types [1]. DPP4 inactivates various oligopeptides composed of proline, hydroxyproline, or alanine as the penultimate residue [2], including incretin hormones secreted by the gastrointestinal tract soon during the postprandial period: glucose-dependent insulinotropic polypeptide (GIP) and glucagon-like peptide-1 (GLP-1). Since these incretins can enhance insulin secretion in a glucose-dependent fashion [3], DPP4 could be considered strictly related to the pathophysiology of type 2 diabetes mellitus [4].

DPP4 is also an adipo-myokine secreted mainly by adipose tissue [5] but also by human myotubes [6]. As an adipokine, DPP4 is mainly released by fully differentiated adipocytes, and its serum levels significantly correlate with adipocyte size [5]. Therefore, it seems to be a marker of visceral obesity, insulin resistance, and metabolic syndrome [7]. On the other hand, the role of DPP4 as a myokine still needs to be understood. Like many adipo-myokines, its tissue concentration may be divergent from its serum level, and distinct auto- and endocrine effects need to be considered [8]. Hypothetically, myokines are essential for muscle metabolism during contraction. On the counterpart, chronic elevation of adipokines may induce adverse effects leading to insulin resistance [8]. There is already evidence that the addition of DPP4 to adipocyte and skeletal and smooth muscle cells inhibits insulin-stimulated Akt phosphorylation, impairing insulin signaling and its action in muscle and fat tissues [5].

Despite the knowledge mentioned above, we still lack studies that associate constitutive DPP4 activity with body composition, measures of adiposity, and also with insulin resistance markers. We suppose that constitutive DPP4 activity might be directly associated not only with fat mass and markers of insulin resistance but also with lean mass. We aimed to investigate if constitutive DPP4 activity correlates with body composition parameters, measures of adiposity, and insulin resistance in subjects with excessive adiposity and different degrees of glucose tolerance. Additionally, we evaluated the relationships between DPP4 activity, adipokines, and gut peptides during the postprandial period.

## 2. Materials and Methods

### 2.1. Subjects

Subjects with excessive adiposity (*n* = 52) were recruited after clinical and laboratory assessments. The study protocol was approved by the Ethics Committee of the University Hospital Pedro Ernesto (CAAE: 24360513.1.0000.5282) and carried out in accordance with the principles of the Declaration of Helsinki. All subjects gave written informed consent.

Participants were subjected to a screening phase before being eligible for the study, which comprised of individual clinical history, physical examination, and weight and height measurements. Fasting plasma glucose (FPG), total cholesterol, high-density lipoprotein, and triglycerides, with a calculated low-density lipoprotein by Friedewald equation, were assessed after 8 h overnight fast. All volunteers without established diabetes mellitus underwent 2 h plasma glucose (PG) after a 75 g oral glucose tolerance test (OGTT).

The inclusion criteria were as follows: men and women aged between 18 and 50 years, with BMI ≥25.0 kg/m^2^, and with or without established diabetes mellitus. Subjects without a preexisting diagnosis of diabetes mellitus were classified into three groups of glucose tolerance, according to the American Diabetes Association (ADA) criteria [9]: (a) normoglycemia/normotolerance (NGT group): FPG <100 mg/dL and 2 h PG in the 75 g OGTT <140 mg/dL; (b) prediabetes (impaired fasting glucose and/or impaired glucose intolerance; pre-DM group): FPG 100 mg/dL to 125 mg/dL and/or 2 h PG in the 75 g OGTT 140 mg/dL to 199 mg/dL; and (c) diabetes mellitus (DM group): FPG ≥126 mg/dL and/or 2 h PG in the 75 g OGTT ≥200 mg/dL. Subjects with a preexisting diagnosis of diabetes mellitus were eligible for the study if exclusively using insulin and/or metformin, and all of them were included in the DM group.

Exclusion criteria were as follows: type 1 diabetes mellitus; the use of any antidiabetic drug, except metformin and insulin (metformin was discontinued for 14 days prior to the exams due to its interference on DPP4 activity [10] and insulin doses were adjusted to avoid hyperglycemia); BMI <25.0 kg/m^2^; uncontrolled chronic diseases, such as arterial hypertension; smoking; severe alcoholism; moderate to severe chronic kidney disease, heart failure, chronic lung disease, and chronic liver disease; fasting serum triglycerides >400 mg/dL; bariatric surgery; the use of immunosuppressant agents; and acute disease at the time of sampling, defined as the presence of moderate to severe malaise, with or without fever.

### 2.2. Study Design

This is a cross-sectional study. On the day of the exams, after 8 h overnight fast, baseline assessments of body composition and anthropometry (weight, height, waist circumference (WC), and hip circumference (HC)) were performed as described below (subsection “body composition and measures of adiposity”). Then, the participants were subjected to three venous blood collections for laboratory measurements (detailed in Section 2.4): baseline (fasting state) and 30 and 60 min after a standardized meal intake (247 kcal, 64.5% carbohydrates, 19.5% protein, 16.0% fat) ingested during up to 5 min.

### 2.3. Body Composition and Measures of Adiposity

We assessed body composition by bioelectrical impedance analysis (BIA, Biodynamic 450 Body Composition Analyzer, BioDynamics®, USA). This method estimated absolute and relative values of fat and lean masses from each participant, who were instructed to avoid exercise and alcohol consumption at least 48 hours prior to the test. BIA was performed in subjects with an empty bladder and wearing light clothing and bare feet.

WC and HC were measured as centimeters at the midway level between the lowest rib margin and the iliac crest and at the widest circumference around the buttocks over the greater trochanters, respectively. The average of two measures of WC and HC was considered for analysis, and waist-to-hip ratio (WHR) was calculated. Height and weight were measured to the nearest 0.5 cm and 0.1 kg using a digital scale with stadiometer (Personal Line 180, Filizola®, Brazil). BMI was calculated as weight in kilograms divided by the square of the height in meters. Body adiposity index (BAI), an index of body adiposity that directly reflects the percentage of body fat, was calculated as follows: BAI = [HC as centimetersI/(height as meter × the square root of height as meter)]–18 [11].

### 2.4. Laboratory Analysis and Surrogate Markers of Insulin Resistance

An intravenous catheter was inserted in the subject's right antecubital vein and kept in place during the exam for venous blood collections. EDTA tubes containing blood samples were centrifuged immediately after collections at 1.300 g for 15 min at 4°C, and plasma samples were clarified by filtration (Millex filter with polyethersulfone membrane, 33 mm, Merck Millipore®, Tullagreen, Ireland), aliquoted, and stored at -80°C.

All laboratory measurements were performed in duplicate as follows: (a) baseline (fasting state): plasma DPP4 activity (colorimetry; sensibility: 0.1 *μ*M/mL/min; intra-assay coefficient of variation [IACV]: <3%); PG (glucose oxidase); GLP-1 (chemiluminescent ELISA; sensibility: 0.14 pM; IACV: <10%; interassay coefficient of variation [IECV]: <15%); sex hormone-binding globulin (SHBG, radioimmune assay); insulin (sensibility: 15 pmol/L), C-peptide (sensibility: 9.5 pg/mL), glucagon (sensibility: 13 pg/mL), leptin (sensibility: 41 pg/mL), and GIP (sensibility: 0.16 pg/mL) (Luminex; Milliplex®, HMHMAG-34K; all IACV and IECV were <10% and <20%, respectively); adiponectin (Luminex; Milliplex®, HADK1MAG-61K; sensibility: 11 pg/mL; IACV: <4%; IECV: <10%); resistin (ELISA; R&D Systems®; DRSN00; sensibility: 0.055 ng/mL; IACV: <6%; IECV: <10%); (b) at 30 and 60 min after meal intake, additional samples for plasma DPP4 activity, PG, insulin, C-peptide, glucagon, GLP-1, GIP, SHBG, leptin, adiponectin, and resistin were collected, prepared, and stored.

The colorimetric method for the measurement of DPP4 activity is based on the degradation of the chromogenic substrate glycyl-prolyl-paranitroanilide (Gly-Pro-pNA, Sigma-Aldrich, Saint Louis, MO, USA). This assay has good precision, linearity, and specificity (at least 95% of the serum Gly-Pro liberating activity can be attributed to DPP4 instead of other dipeptidyl peptidases) [12]. The results were expressed as p-nitroaniline/mL/min. FPG and insulin were used to calculate the homeostasis model assessment of insulin resistance (HOMA-IR) and the quantitative insulin sensitivity check index (QUICKI) as follows: HOMA-IR = insulin (*μ*UI/mL) x FPG (mmol/mL)/22.5 [13] and QUICKI = 1/[log(insulin, *μ*UI/mL) + log(FPG, mg/dL)] [14]. Since low SHBG level is a recognized marker of insulin resistance [15], we used SHBG, fasting insulin, and HOMA-IR to evaluate insulin resistance and QUICKI as a surrogate marker of insulin sensitivity.

### 2.5. Statistical Analysis

We used GraphPad Prism® 5 (GraphPad Software Inc., San Diego, CA, USA) and STATISTICA® 7.0 (StatSoft Inc., Tulsa, OK, USA) for statistical analysis. Gaussian distribution was checked and we expressed parametric and nonparametric variables as mean±SD and median [1st–3rd quartiles], respectively. We used one-way analysis of variance (ANOVA) with Dunn's multiple comparison test to compare groups with normally distributed data, and Fisher exact test was used for categorical data analysis. In the pooled analysis, linear correlations between DPP4 activity and the other variables were conducted. In a step further, multiple regression analysis was also performed to test whether some body composition–anthropometric–laboratory variables could influence DPP4 activity. Values of *P* < 0.05 were considered statistically significant.

## 3. Results

Fifty-two subjects with excessive adiposity, aged 38.9 ± 8.36 years and with BMI 29.09 ± 2.55 kg/m^2^, were included and almost half of them (48.0%) were women. Six subjects in DM group had a preexisting diagnosis of diabetes mellitus and did not undergo OGTT. Among those without a previous diagnosis, two had both FPG, and 2 h PG in the 75 g OGTT criteria, and two had only one of each criterion. At baseline, we noticed significant differences in gender, BMI, BAI, absolute fat mass, FPG, and GIP among NGT, pre-DM, and DM groups (see Table 1). Fasting insulin and HOMA-IR were higher and QUICKI was lower in pre-DM compared to NGT group (DM group was not included in this analysis since some of these patients were using exogenous insulin). Of note, there was no difference in DPP4 activity among groups. We calculated the AUC of DPP4 activity, insulin, C-peptide, glucagon, GLP-1, GIP, leptin, adiponectin, and resistin during meal test and investigated possible differences that could be expressed only after this stimulus. We found differences among groups only for PG_AUC_ (6311.59 ± 859.54 vs. 7170 ± 731.77 vs. 10879.5 ± 4421.83 for NGT, pre-DM, and DM groups, respectively; *P* < 0.0001) and GIP_AUC_ (5513 [4411.75–7242.75] vs. 7321 [5450–8977] vs. 7671 [6033.75–9989.25] for the same groups, respectively; *P* = 0.032). No other difference was observed in this analysis, as evidenced in the Supplementary Material (see Table S1).

**Figure 1:**
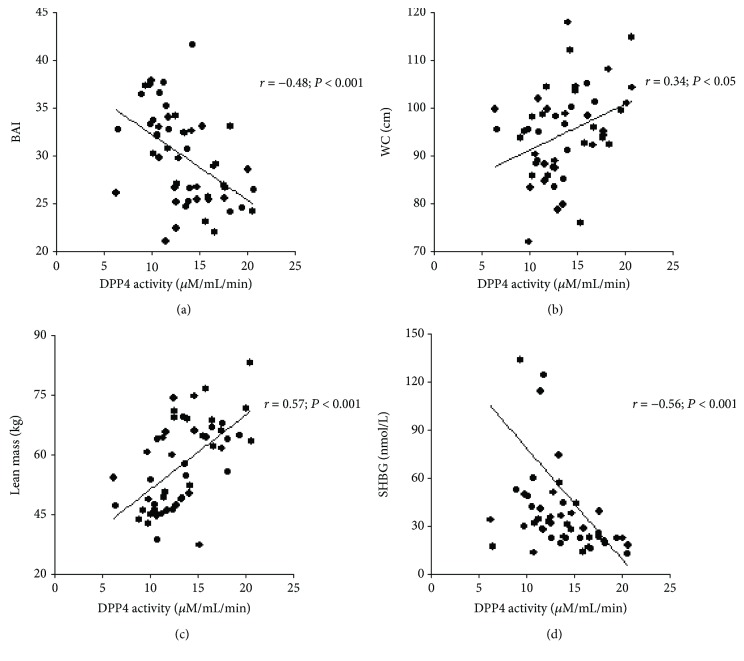
Scatter plots demonstrating bivariate correlations between DPP4 activity and other variables in the pooled group. DPP4 activity was correlated with (a) BAI, (b) WC, (c) absolute lean mass, and (d) SHBG (*n* = 52). In Figure 1(d), four data points are outside the *y*-axis limits. DPP4: dipeptidyl peptidase 4; BAI: body adiposity index; WC: waist circumference; SHBG: sex hormone-binding globulin.

To test our hypothesis, we correlated DPP4 activity with anthropometry, body composition variables, and insulin resistance markers in the pooled sample.  Table 2 presents the observed correlations. Of interest, we observed some associations between baseline DPP4 activity and anthropometric measures and derived indexes as follows: direct correlations with weight, WC, and WHR and an inverse association with BAI. Considering body composition variables, baseline DPP4 activity was correlated positively with absolute and percent values of total cell mass and lean mass and inversely with the percentage of fat mass. No correlation between baseline DPP4 activity and absolute fat mass was found. Additionally, there were significant relationships between baseline DPP4 activity and all insulin resistance markers herein tested, with the exception of the triglycerides-to-high-density lipoprotein ratio. As noted, we found positive correlations with insulin and HOMA-IR and negative correlations with SHBG and QUICKI. Some of these correlations are shown in Figure 1.

Since body composition parameters, measures of adiposity, and SHBG levels are all influenced by gender, we performed sex-specific analysis and observed that some baseline DPP4 activity correlations previously found remained for men but not for women as follows: WHR (*r* = 0.44, *P* = 0.024), insulin levels (*r* = 0.40, *P* = 0.031), and SHBG (*r* = −0.40, *P* = 0.038). By including data from postprandial period of all noninsulinized subjects (*n* = 46), we also observed positive linear correlations of DPP4 activity_AUC_ with insulin_AUC_ (*r* = 0.36, *P* = 0.018) and C-peptide_AUC_ (*r* = 0.28, *P* = 0.047) (see Table 3), which suggests DPP4 activity is associated with insulin resistance also after meal challenge.

We observed other interesting associations between DPP4 activity and biochemical variables. At baseline, DPP4 activity was associated with total cholesterol (*r* = 0.31, *P* = 0.022) and LDL (*r* = 0.36, *P* = 0.008). Baseline DPP4 activity was also associated with leptin (*r* = −0.31, *P* = 0.023) and resistin (*r* = −0.32, *P* = 0.020), and there was a trend toward a correlation with adiponectin (*r* = −0.26, *P* = 0.059) (see Table 2). This same pattern was observed for DPP4 activity_AUC_; it was correlated with leptin_AUC_ (*r* = −0.31, *P* = 0.021) and resistin_AUC_ (*r* = −0.32, *P* = 0.021) and there was a trend toward an association with adiponectin_AUC_ (*r* = −0.27, *P* = 0.051) (see Table 3).

In the next step, we tested whether some body composition–anthropometric–laboratory variables could, together, influence the DPP4 activity (see Table 4). Our results on multiple regression analysis showed that BAI, WHR, percent lean mass, HOMA-IR, and leptin influenced DPP4 activity and explained approximately 26% of its variance. BAI and HOMA-IR exerted the more significant effect on DPP4 activity, reassuring the existence of a role derived from body fat and insulin resistance in the plasma DPP4 activity of subjects with excessive adiposity.

## 4. Discussion

Beyond DPP4 enzyme role in the pathophysiology of type 2 diabetes, this protein is also an adipo-myokine [5, 6] and, therefore, it is supposed to be related to lean and fat masses. However, as far as we know, there are very few studies [16–18] assessing the relationship between DPP4 activity and anthropometric/body composition measures and all of them evaluated healthy people. In the present study, interesting original findings were observed regarding DPP4 activity associations with some anthropometric, body composition, and laboratory variables in subjects with excessive adiposity and different stages of glucose tolerance.

At first, baseline DPP4 activity was positively associated with weight and total cell mass, which was expected, since DPP4 is an adipo-myokine [5, 6] and body weight is mostly composed of fat and lean masses [19]. Furthermore, DPP4 activity was associated with all insulin resistance markers herein tested (i.e., we found direct associations with fasting insulin and HOMA-IR and inverse associations with SHBG and QUICKI). By testing it in a sex-specific fashion, the observed results were not kept unchanged, except for fasting insulin and SHBG in men. Possibly, the above finding may have occurred because of low statistical power, since insulinized subjects were excluded from almost all of these subanalyses. We should emphasize that DPP4 activity_AUC_ was also correlated with insulin_AUC_. Altogether, these results indicate a direct association between DPP4 activity and insulin resistance, which is consistent with two studies that found positive associations between DPP4 activity and HOMA-IR in Chinese populations without diabetes [20, 21]. Interestingly, there was no difference in DPP4 activity among groups with different degrees of glucose tolerance, despite the consistent association of DPP4 activity with insulin resistance. Maybe it occurred because multiple factors beyond insulin resistance determine glucose tolerance.

We also noted that baseline DPP4 activity was inversely correlated with baseline resistin, and this same pattern was reproduced on the observed correlation between DPP4 activity_AUC_ and resistin_AUC_. As its name expresses, resistin appears to have a role in insulin resistance, but some evidence on this issue is paradoxical and hard to explain [22, 23]. For example, factors that are known to be related to insulin resistance, such as hyperinsulinemia and TNF-*α*, counter-regulate expression of resistin mRNA and protein in cultured adipocytes [23]. Considering our findings that DPP4 activity and insulin levels were positively associated, the latter might be a confounder for the negative correlations between baseline and AUC of DPP4 activity and resistin, respectively, although a causal relationship cannot be excluded.

DPP4 activity was positively associated with WC and WHR, both known as measures of central adiposity and visceral adipose tissue (VAT) as well, but it was inversely correlated with (a) the percentage of fat mass; (b) the BAI, an index of general body adiposity [11]; and (c) leptin, an adipokine mainly released by subcutaneous adipose tissue (SAT) [24]. Moreover, there were some trends toward inverse correlations between DPP4 activity and adiponectin (*P* = 0.05), an adipokine with insulin sensitizer properties that is inversely related to general obesity and central fat distribution [25] and also between DPP4 activity_AUC_ and adiponectin_AUC_ (*P* = 0.05) during the postprandial period. Altogether, these findings suggest that VAT may be a determinant of higher DPP4 activity and SAT may have a negative impact on it. The hypothesis is partly supported by some bench studies [5, 7], by a clinical study evaluating DPP4 levels in patients with familial partial lipodystrophy type 2 (an inherited disease characterized by lack of SAT and fat deposition in VAT and ectopic sites) [26] and also by the knowledge that SAT is a determinant of metabolic health [27]. Curiously, a meta-analysis of ten randomized controlled trials evaluating the impact of DPP4 inhibitors on serum adiponectin of patients with type 2 diabetes confirmed significantly elevated adiponectin levels after the pharmacological inhibition of DPP4 [28].

Neidert and coworkers [16] evaluated the correlations between DPP4 activity and body composition variables in healthy lean subjects and provided the first evidence of a positive association between DPP4 activity and absolute lean mass. Additionally, they found that (a) DPP4 activity was negatively correlated with absolute fat mass and gynoid fat and (b) there was no significant relationship between DPP4 activity and BMI [16]. Although we did not find an association between DPP4 activity and BMI either, these results differ from those of three studies involving people without diabetes [17, 20, 21], in which mild but significant positive correlations were found. The association of DPP4 activity with lean mass reinforces the characterization of DPP4 as a myokine [6]. Since lean mass is inversely related to insulin resistance [29], our findings that DPP4 activity is positively associated with lean mass and also with measures of central obesity and insulin resistance might seem paradoxical. However, it seems to be the case for many adipo-myokines [8]. For example, the interleukin-6 (IL-6) has different influences depending on its source, time of action, and target tissue [8], and herein, we hypothesize a parallel mode of action of the DPP4 tested. Finally, we evidenced that BAI, WHR, percent lean mass, HOMA-IR, and leptin altogether influenced the plasma DPP4 activity, with BAI and HOMA-IR exerting more significant effect on it.

Our study has strengths and limitations. Absolute majority of studies assessed DPP4 levels instead of its activity, and, as far as we know, this is the first study evaluating DPP4 activity and its relation to measures of adiposity, body composition, and insulin resistance in subjects with excessive adiposity and different stages of glucose tolerance. Moreover, we assessed adipokines and other biochemical variables to understand the results of our primary analysis better. Limitations of the study are associated with its cross-sectional design, implying it is explicitly correlational and it does not directly demonstrate causality, and with the small sample size, limiting the power of our subgroup analysis. Therefore, we highlight the results cannot be generalized since the study sample is limited and involves only subjects with excessive adiposity.

## 5. Conclusions

Constitutive DPP4 activity is positively associated with muscle mass, measures of central adiposity, and insulin resistance, but it is inversely associated with indexes of general body adiposity, which is consistent with the hypothesis that VAT is a determinant of higher DPP4 activity and SAT may have a negative impact on it. Additionally, DPP4 activity seems to be influenced by some body composition–anthropometric–laboratory variables, suggesting this adipo-myokine activity could be influenced by body composition aspects of subjects with excessive adiposity and different stages of glucose tolerance.

## Figures and Tables

**Table 1:** Clinical and biochemical characteristics of subjects at baseline.

	All groups (*n* = 52)	NGT group (*n* = 22)	Pre-DM group (*n* = 20)	DM group (*n* = 10)	*P* value
Age (years)	38.9 ± 8.36	38 ± 8.21	39.65 ± 9.18	39.4 ± 7.56	0.657
Female [*n*, (%)]	25 (48%)	8 (36.3%)	11 (55%)	6 (60%)	**<0.01**
BMI (kg/m^2^)	29.09 ± 2.55	27.52 ± 1.63	30.74 ± 2.74‡	29.23 ± 1.63	**0.0004**
BAI	29.93 ± 4.84	27.95 ± 4.52	31.79 ± 4.93^∗^	30.56 ± 4.03	**0.023**
WC (cm)	94.85 ± 9.32	90.95 ± 8.93	77.29 ± 7.7	98.55 ± 10.77	0.073
HC (cm)	104.9 ± 5.8	102.8 ± 4.62	107.7 ± 6.57	103.7 ± 4.51	0.055
WHR	0.9 ± 0.08	0.88 ± 0.08	0.9 ± 0.07	0.95 ± 0.09	0.164
Total cell mass (kg)	28.8 ± 6.15	29.14 ± 5.92	28.98 ± 6.31	27.7 ± 6.82	0.685
Total cell mass (%)	33.65 [31.28–35.98]	34.95 [33.48–37.75]	32.75 [30.55–35.3]	32.6 [30.58–36.73]	0.055
Fat mass (kg)	25.81 ± 4.9	23.25 ± 3.69	28.73 ± 4.83†	25.63 ± 4.47	**0.001**
Fat mass (%)	31.22 ± 5.7	29.05 ± 5.87	33.39 ± 5.27	31.66 ± 4.82	0.066
Lean mass (kg)	57.68 ± 11.04	58.07 ± 11.14	58.22 ± 11.74	55.74 ± 10.26	0.848
Lean mass (%)	68.78 ± 5.7	70.95 ± 5.87	66.62 ± 5.27	68.34 ± 4.82	0.066
HOMA-IR	1.99 ± 1.66^a^	1.24 ± 0.82	2.82 ± 1.96	—	**0.001**
QUICKI	0.35 [0.33–0.38]^a^	0.33 [0.31–0.35]	0.3 [0.29–0.36]	—	**0.001**
SHBG (nmol/L)	31.63 [22.47–49.42]	37.05 [22.68–51.37]	28.96 [22.51–46.77]	28.28 [17.75–60.57]	0.476
THR	2.16 [1.78–3.52]	2.16 [1.72–3.66]	2.62 [1.49–3.55]	2 [1.82–3.38]	0.983
DPP4 activity (*μ*M/mL/min)	13.46 ± 3.41	13.79 ± 2.69	13.28 ± 3.64	13.1 ± 4.56	0.638
FPG (mg/dL)	106.3 ± 38.73	87.45 ± 8.4	104.7 ± 9.7‡	151 ± 71.01‡	**<0.0001**
Insulin (pmol/L)	62.86 ± 48.31^a^	40.87 ± 27.98	74.74 ± 49.35	—	**0.008**
C-Peptide (pg/mL)	691.3 ± 330.7^a^	629.6 ± 327.3	759.2 ± 329.2	—	0.208
Glucagon (pg/mL)	13.28 [15.31–51.01]	9.76 [5.08–26.76]	24.5 [10.42–29.7]	16.42 [5.62–31.99]	0.152
GLP-1 (pM)	0.73 [0.11–1.28]	0.66 [0.11–1.49]	0.81 [0.37–1.3]	0.61 [0.11–1.06]	0.444
GIP (pg/mL)	23.63 [15.31–51.01]	19.45 [12.22–25.94]	27.88 [18.64–48.81]	49.3 [31.15–67.44]^∗^	**0.021**
Leptin (pg/mL)	6278 [3766–11650]	4231 [2496–9251]	7697 [4790–12735]	6117 [4284–13365]	0.077
Adiponectin (pg/mL)	12.67 [8.25–17.17]	11.92 [8.76–16.2]	13.55 [6.01–21.36]	11.76 [7.14–23.99]	0.888
Resistin (ng/mL)	6.34 [5.23–8]	6.74 [5.28–13.73]	6.32 [5.19–10.64]	6.3 [4.63–10.44]	0.913
Total cholesterol (mg/dL)	200.5 [170–234.3]	200.5 [175.3–236]	201.5 [170.3–231]	194.5 [145.5–253]	0.920
HDL (mg/dL)	49.5 [41–59]	48 [40.75–58.25]	53 [40–60]	49.5 [40.5–63]	0.831
LDL (mg/dL)	126.5 [96.25–153.5]	127.5 [108.5–153.8]	119.5 [96–155.8]	129 [79–160.3]	0.895
TG (mg/dL)	133.1 ± 60.12	131.1 ± 62.08	138.3 ± 65.6	127.4 ± 47.93	0.969
*Screening phase*					
FPG (mg/dL)	99.5 [92.25–113]	92 [85.5–96.25]	106.5 [100.8–112.3] ‡	169 [152–267.8]‡§	**<0.0001**
2 h PG in the 75 g OGTT (mg/dL)	127.3 ± 39.11	102.5 ± 21.1	138.8 ± 28.42†	206.8 ± 29.2‡^b^	**<0.0001**
Total cholesterol (mg/dL)	198.3 ± 50.81	194.7 ± 40.52	210.5 ± 58.53	181.9 ± 53.89	0.524
HDL (mg/dL)	56.04 ± 16	48.27 ± 11.68	62.25 ± 16.22^∗^	60.7 ± 17.88	**0.009**
LDL (mg/dL)	115.4 ± 41.62	121.8 ± 39.06	117.6 ± 47.34	97 ± 32.17	0.248
TG (mg/dL)	119.5 [83–170.8]	116.5 [83–155.8]	141.5 [82.25–199.8]	106 [82.25–162.5]	0.521

Data are presented as mean±SD, median [1st–3rd quartiles], or *n* (%). ^∗^ *P* < 0.05, †*P* < 0.01, and ‡*P* < 0.001 in comparison to NGT group. §*P* < 0.05 in comparison to pre-DM group. ^a^Pooled data of NGT and pre-DM groups. ^b^Only 04 subjects performed OGTT in the DM group. NGT: normoglycemia; pre-DM: prediabetes; DM: diabetes mellitus; BMI: body mass index; BAI: body adiposity index; WC: waist circumference; HC: hip circumference; WHR: waist-to-hip ratio; HOMA-IR: homeostasis model assessment to quantify insulin resistance; QUICKI: quantitative insulin sensitivity check index; SHBG: sex hormone-binding globulin; THR: triglycerides-to-high-density lipoprotein ratio; DPP4: dipeptidyl peptidase 4; FPG: fasting plasma glucose; GLP-1: glucagon-like peptide-1; GIP: glucose-dependent insulinotropic polypeptide; HDL: high-density lipoprotein; LDL: low-density lipoprotein; TG: triglycerides; PG: plasma glucose; OGTT: oral glucose tolerance test.

**Table 2:** Linear correlations between DPP4 activity and variables of the pooled group at baseline (*n* = 52).

	*r* value	*P* value
Age	-0.17	0.214
Weight	0.56	**<0.0001**
BMI	0.22	0.116
BAI	-0.48	**0.0003**
WC	0.34	**0.011**
HC	-0.03	0.809
WHR	0.35	**0.009**
Total cell mass (kg)	0.58	**<0.0001**
Total cell mass (%)	0.47	**0.0004**
Fat mass (kg)	0.11	0.432
Fat mass (%)	-0.33	**0.014**
Lean mass (kg)	0.57	**<0.0001**
Lean mass (%)	0.33	**0.014**
HOMA-IR^a^	0.29	**0.046**
QUICKI^a^	-0.29	**0.046**
SHBG	-0.56	**<0.0001**
THR	0.25	0.063
FPG	-0.01	0.911
Insulin^a^	0.32	**0.025**
C-Peptide^a^	0.25	0.085
Glucagon	0.18	0.192
GLP-1	0.008	0.952
GIP	0.06	0.672
Leptin	-0.31	**0.023**
Adiponectin	-0.26	0.059
Resistin	-0.32	**0.020**
Total cholesterol	0.31	**0.022**
HDL	-0.1	0.468
LDL	0.36	**0.008**
TG	0.22	0.111

^a^All six insulinized subjects from DM group were excluded from analysis. DM: diabetes mellitus; DPP4: dipeptidyl peptidase 4; BMI: body mass index; BAI: body adiposity index; WC: waist circumference; HC: hip circumference; WHR: waist-to-hip ratio; HOMA-IR: homeostasis model assessment to quantify insulin resistance; QUICKI: quantitative insulin sensitivity check index; SHBG: sex hormone-binding globulin; THR: triglycerides-to-high-density lipoprotein ratio; FPG: fasting plasma glucose; GLP-1: glucagon-like peptide-1; GIP: glucose-dependent insulinotropic polypeptide; HDL: high-density lipoprotein; LDL: low-density lipoprotein; TG: triglycerides.

**Table 3:** Linear correlations between DPP4 activity_AUC_ and the AUC of biochemical variables of the pooled group (*n* = 52).

	*r* value	*P* value
PG	-0.06	0.642
Insulin^a^	0.35	**0.018**
C-Peptide^a^	0.28	**0.047**
Glucagon	0.22	0.102
GLP-1	-0.13	0.348
GIP	0.23	0.098
Leptin	-0.31	**0.021**
Adiponectin	-0.27	0.051
Resistin	-0.32	**0.017**

^a^All six insulinized subjects from DM group were excluded from analysis (*n* = 46). DM: diabetes mellitus; DPP4: dipeptidyl peptidase 4; PG: plasma glucose; GLP-1: glucagon-like peptide-1; GIP: glucose-dependent insulinotropic polypeptide.

**Table 4:** Multiple regression analysis of the pooled group.

Model	*β*	*P* value	Adjusted *R* ^2^	*P* value
			0.265	<0.0001
BAI	**-0.473**	**0.0002**		
HOMA-IR	**0.291**	**0.019**		

Forward selection; *F* = 10.199; *P* = 0.0001. DPP4 activity was considered as the dependent variable and BAI, WHR, percent lean mass, HOMA-IR, and leptin as independent variables in the model. Mean substitution strategy was adopted to HOMA-IR values of six insulinized subjects. DPP4: dipeptidyl peptidase 4; BAI: body adiposity index; WHR: waist-to-hip ratio; HOMA-IR: homeostasis model assessment to quantify insulin resistance.

## Data Availability

The datasets used and/or analyzed during the current study are available from the corresponding author on reasonable request. All data generated or analyzed during this study are included in this published article and its supplementary information file.

## Supplementary Materials

Supplementary Materials Table S1: intergroup comparisons between the AUC of biochemical variables of the NGT, pre-DM, and DM groups.
