# Supplementary material for: Dipeptidyl Peptidase 4 Activity Is Related to Body Composition, Measures of Adiposity, and Insulin Resistance in Subjects with Excessive Adiposity and Different Degrees of Glucose Tolerance
Source: J Diabetes Res. 2019 Feb 11;2019:5238013. doi: 10.1155/2019/5238013 (PMC6388345; doi:10.1155/2019/5238013)
Supplement: Supplementary Materials — Table S1: intergroup comparisons between the AUC of biochemical variables of the NGT, pre-DM, and DM groups. [file 5238013.f1.docx]

**Dipeptidyl peptidase 4 activity is related to body composition, measures of adiposity, and insulin resistance in subjects with excessive adiposity and different degrees of glucose tolerance.**

Wellington S. Silva Júnior, Maria das Graças C. Souza, José F. Nogueira Neto, Eliete Bouskela, and Luiz Guilherme Kraemer-Aguiar.

**Table S1. Intergroup comparisons between the AUC of biochemical variables of the NGT, Pre-DM, and DM groups.**

|  | All groups  (n = 52) | NGT group  (n = 22) | Pre-DM group  (n = 20) | DM group  (n = 10) | P-value |
| --- | --- | --- | --- | --- | --- |
| DPP4 activity | 773.99 ± 190.74 | 792.53 ± 156.76 | 764.78 ± 192.3 | 751.63 ± 263.01 | 0.659 |
| PG | 7520.19 ± 2616.31 | 6311.59 ± 859.54 | 7170 ± 731.77* | 10879.5 ± 4421.83‡ | <0.0001 |
| Insulin | 68910 [56306.25–88308.75]^a^ | 61312.5 [46788.75–86925] | 77985 [65471.25–96873.75] | ― | 0.051 |
| C-peptide | 72975 [57540–101437.5]^a^ | 72247.5 [55252.5–87465] | 87555 [62512.5–103717.5] | ― | 0.251 |
| Glucagon | 979.65 [487.25–1179] | 741 [487.25–1179] | 1596 [570.6–1807.5] | 1159.65 [710.52–1624.25] | 0.079 |
| GLP-1 | 113.53 ± 80.83 | 107.21 ± 84.23 | 116.58 ± 78.81 | 121.34 ± 84.69 | 0.749 |
| GIP | 6620 [5160.25–8684.25] | 5513 [4411.75–7242.75] | 7321 [5450–8977] | 7671 [6033.75–9989.25] | 0.032 |
| Leptin | 337260 [205912.5–613897.5] | 231277.5 [145492.5–491028.75] | 448957.5 [292642.5–666243.75] | 318015 [233805–636330] | 0.104 |
| Adiponectin | 712.75 [492.65–1005.32] | 665.55 [509.8–865.15] | 807.75 [361.72–1189] | 700.9 [413.4–1122.27] | 0.818 |
| Resistin | 377.3 [298.3–465.5] | 387.1 [297.4–458.8] | 371.8 [302.3–487.5] | 367.2 [265.1–526.3] | 0.964 |

Data are presented as mean ± SD or median [1st–3rd quartiles]. *P<0.05 and ‡P<0.001 in comparison to NGT group. ^a^Pooled data of NGT and Pre-DM groups. NGT: normoglycemia; Pre-DM: prediabetes; DM: diabetes mellitus; DPP4: dipeptidyl peptidase 4; PG: plasma glucose; GLP-1: glucagon-like peptide-1; GIP: glucose-dependent insulinotropic polypeptide.
